# AST/ALT ratio predicts the functional severity of chronic heart failure with reduced left ventricular ejection fraction

**DOI:** 10.1186/s13104-020-05031-3

**Published:** 2020-03-24

**Authors:** Mohammed Ewid, Hossam Sherif, Abdulaziz S. Allihimy, Shaima A. Alharbi, Dawood A. Aldrewesh, Sarah A. Alkuraydis, Rami Abazid

**Affiliations:** 1 Faculty of Medicine, Sulaiman AlRajhi University, P.O. Box 777, Al Bukairyah Qassim, 51941 Saudi Arabia; 2 Internal Medicine Department, Faculty of Medicine, Cairo University, Cairo, 11562 Egypt; 3 Medical Student, College of Medicine, Qassim University, Qassim, Saudi Arabia; 4 Critical Care Medicine Department, Faculty of Medicine, Cairo University, Cairo, Egypt; 5 Prince Sultan Cardiac Center, Ministry of Health, Buraidah Qassim, Saudi Arabia; 6 Department of Nuclear Medicine, London Health Sciences Center, 800 Commissioners Road East, PO Box 5010, London, ON N6A 5W9 Canada

**Keywords:** AST/ALT ratio, Heart failure, Hepatic fibrosis

## Abstract

**Objective:**

Despite previous research that focused on liver transaminases as predictors of cardiovascular disease, there has been limited research evaluating the predictive value of AST/ALT ratio in patients with heart failure. We aimed to investigate AST/ALT ratio as an indicator of the functional severity in chronic heart failure with reduced left ventricular ejection fraction.

**Results:**

Overall, 105 patients previously diagnosed with HFrEF from Buraidah-Al Qassim province, Saudi Arabia were included in this retrospective cross-sectional study. Data on study variables, including demographic data, left ventricular ejection fraction, NYHA class, and AST/ALT ratio, were collected from patients’ records. The patients were divided into two groups, namely group-1 (AST/ALT ratio < 1) and group-2 (AST/ALT ratio ≥ 1), to identify any differences in their cardiac function profiles. NYHA class and NT-proBNP were higher and LVEF was lower in group-2 than in group-1. We found a mild significant correlation between AST/ALT ratio and APRI, FIB-4 score, NYHA-class, and LVEF (r = 0.2, 0.25, 0.26, and − 0.24, respectively; P < 0.05). Multivariate linear regression analysis model and ROC curve showed that AST/ALT ratio could independently predict HFrEF functional severity with a best cut-off value of 0.9, sensitivity of 43.6%, and specificity of 81.4%.

## Introduction

Medical care has greatly advanced over the last three decades. However; heart failure remains a major cause of morbidity and mortality, with a general prevalence rate of approximately 2% and an abrupt rise to 9% in those aged ≥ 65 years [1].

Additionally, the prevalence of heart failure is increasing due to an increase in cardiovascular risk factors and increased life expectancy [2].

Established cardiovascular biomarkers include N-terminal pro-brain natriuretic peptide (NT-proBNP) [3] and troponin [4], while emerging biomarkers include miRNAs [5], mimecan [6], and orexin [7]. However, none of these biomarkers are routinely measured, indicating the need for new feasible and reproducible biomarkers that help in screening the functional severity in heart failure patients with reduced ejection fraction (HFrEF).

Consequently, research is still continuing to improve risk stratification tools for cardiovascular disease (CVD), including those specific for chronic heart failure.

Multiple theories have attempted to explain the cardiovascular-hepatic relationship that has been documented with abnormal liver function test results in patients with CVD, including heart failure [8], ischemic heart disease [9], and atherosclerosis [10].

Hepatic fibrosis is no longer considered an isolated hepatic disease on the basis of its association with multisystem dysfunction, including CVD [11]. Non-invasive indices were developed for early detection and monitoring of hepatic fibrosis [12]. The validated indices include the Aspartate aminotransferase (AST)/Alanine aminotransferase (ALT) ratio [13], AST to platelet ratio index (APRI) [14], Fibrosis-4 score (FIB-4) [15], and non-alcoholic fatty liver disease (NAFLD) fibrosis score [16].

Previous reports highlighted the value of non-invasive hepatic fibrosis indices as prognostic tools in hepatic patients [17]. However, there is little research evaluating these indices in HFrEF patients.

In our study, we have focused on the role of AST/ALT ratio in predicting the functional severity of HFrEF.

## Main text

### Methods

#### Study design and participants

This retrospective cross-sectional study included 105 patients recruited from the heart failure outpatient clinic of Prince Sultan Cardiac Center Qassim (PSCCQ), Buraidah, Saudi Arabia.

All recruited patients had HFrEF according to American College of Cardiology heart failure diagnostic criteria, evidenced by left ventricular ejection fraction (LVEF) ≤ 40% [18]; we excluded those with any of the following: primary liver disease, sepsis, shock, malignancy, pregnancy, or renal failure.

#### Demographic data

The following variables were collected: age, sex, duration of heart failure, and cardiovascular risk factors including; obesity, diabetes mellitus, smoking, and hypertension.

#### Laboratory investigations and calculation of liver fibrosis indices

Complete blood count, liver function tests, kidney function tests, thyroid profile, and NT-proBNP levels were recorded.

We calculated the following standard non-invasive hepatic fibrosis indices:

- AST/ALT ratio [13].
- AST to platelet ratio index (APRI) [14] = ([AST/top normal AST]/platelet count [10^9^ /liter]) × 100.
- Fibrosis-4 (FIB-4) [15] = (Age × AST)/(Platelet count × ALT square root).

#### Transthoracic echocardiography

Transthoracic echocardiography was performed according to the recommendations of both the American Society of Echocardiography and European Association of Cardiovascular Imaging.

The modified Simpson’s rule was used to estimate left ventricular (LV) volumes and measure the ejection fraction [19].

#### Estimation of systolic pulmonary arterial pressure (SPAP)

SPAP was estimated using the modified Bernoulli formula (SPAP [mmHg] = 4 × tricuspid regurgitation velocity^2^ + right atrial pressure) [20].

#### Classification of patients according to the AST/ALT ratio

Based on literature review, an AST/ALT ratio ≥ 1 is highly specific and predictive of liver cirrhosis in patients with chronic HCV infection [13]; we used the same cut-off value to classify our patients into two groups:

- Group-1: AST/ALT ratio < 1.0
- Group-2: AST/ALT ratio ≥ 1.0

We adopted patients’ laboratory values, New York Heart Association (NYHA) functional Class and LVEF measurements that were performed after their first interview in the outpatient clinic.

#### Statistical methods

The statistical package MedCalc version 19.0.5 was used for all statistical analyses. Quantitative data are presented as mean ± standard deviation, and qualitative data are presented as percentages. Comparisons between groups were made using the Mann–Whitney test. Correlations between variables were analyzed using Spearman’s rank correlation coefficient. Multivariate linear regression was used to investigate the independent predictability of AST/ALT ratio for LVEF percentage [LVEF (%)].

## Results

This study included 105 patients (50 ± 14.71, 16–88 years; 68 men). Both study groups showed comparable results; however, Group-2 showed a less favorable cardiac profile. NYHA class and NT-proBNP were higher and LVEF was lower in group-2 than in group-1 (Table 1).

**Table 1:** Descriptive data of subjects with AST/ALT ratio < 1 and ≥ 1

Parameter	Group-1 (58 patients) [AST/ALT ratio < 1]	Group-2 (47 patients) [AST/ALT ratio ≥ 1]	P-value
Age (year)	50.03 ± 15.25 (16–82)	50.17 ± 14.18 (21–88)	0.915
Gender (n, %)	44 males (75%)	24 males (51%)	0.009
Heart failure Duration (year)	5.43 ± 2.46 (2–12)	4.55 ± 3.09 (1–13)	0.021
NYHA–class	2.12 ± 1.04 (1–4)	2.49 ± 0.86 (1–4)	0.042
Diabetes mellitus (n, %)	37 (63.8%)	26 (55.32%)	0.052
Hypertension (n, %)	33 (56.9%)	25 (53.2%)	0.632
Smoking (n, %)	10 (17.24%)	11(23.4%)	0.493
BMI (kg/m^2^)	28.72 ± 5.8 (16–40)	30.33 ± 9.42 (17.6–70)	0.609
Systolic BP (mmHg)	123.19 ± 20.19 (90–185)	127.02 ± 17.5 (100–171)	0.248
Diastolic BP (mmHg)	73 ± 11.57 (52–99)	73.68 ± 10.63 (50–111)	0.701
Hemoglobin (g/dL)	13.65 ± 1.76 (9.2–17.7)	12.86 ± 2.18 (7.5–16.3)	0.086
Platelets (× 10^9^/L)	267.68 ± 72.25 (137–422)	299.71 ± 94.94 (149–588)	0.180
Fasting blood sugar (mmol/L)	15.62 ± 27.62 (4.38–140)	10.24 ± 6.44 (4.82–24.8)	0.874
Potassium^+^ (mEq/L)	4.2 ± 0.52 (3.1–5.8)	4.1 ± 0.58 (2.8–5.7)	0.415
Sodium^+^ (mEq/L)	136.96 ± 4.43 (127–146)	135.98 ± 3.83 (127–143)	0.261
Serum creatinine (mmol/L)	109.57 ± 80.95 (50–581)	114.86 ± 97.72 (57–538)	0.741
Blood urea (mmol/L)	7 ± 3.36 (2.7–19)	7.34 ± 5.27 (2.8–30.6)	0.588
Total cholesterol (mmol/L)	3.99 ± 1.13 (1.06–5.86)	4.35 ± 1.64 (1.2–7.8)	0.373
HDL cholesterol (mmol/L)	0.93 ± 0.25 (0.53–1.39)	0.99 ± 0.35 (0.25–1.68)	0.459
Triglycerides (mmol/L)	2.08 ± 1.36 (0.51–6.36)	1.57 ± 0.99 (0.64–4.94)	0.068
T3 (nmol/L)	5.25 ± 2.75 (1.11–16.95)	6.87 ± 4.78 (4.07–17.67)	0.655
T4 (nmol/L)	13.64 ± 5.15 (1.62–20.7)	13.75 ± 4.46 (3.71–19.99)	0.981
TSH (mIU/L)	9.15 ± 32.54 (1–193)	6.6 ± 10.05 (0.19–48)	0.899
Bilirubin (mg/dL)	11.88 ± 11.63 (3–67.9)	12.75 ± 12.2 (0.7–64)	0.722
Albumin (g/dL)	36.02 ± 4.51 (24.2–45.4)	33.68 ± 8.61 (38–44.2)	0.492
aPTT (s)	38.08 ± 20.72 (16–126)	35.38 ± 13.57 (18–84.3)	0.549
INR	1.15 ± 0.15 (1–1.6)	1.22 ± 47 (0.94–3.7)	0.737
AST (IU/L)	32.03 ± 50.31 (10–351)	75.17 ± 163.59 (7–763)	0.195
ALT (IU/L)	52.05 ± 71.05 (15–520)	25.57 ± 24.37 (4–121)	0.001
GGT (U/L)	89.67 ± 101.8 (6–203)	25.48 ± 19.33 (6–52)	0.479
ALP (U/L)	102.38 ± 57.81 (48–385)	98.89 ± 53.14 (34–276)	0.449
AST/ALT ratio	0.63 ± 0.19 (0.16–0.94)	1.3 ± 0.25 (0.95–1.85)	0.001
APRI index	0.31 ± 0.41 (0.09–2.8)	0.74 ± 1.76 (0.07–8.03)	0.877
FIB-4 score	0.8 ± 0.43 (0.17–1.96)	1.1 ± 0.81 (0.3–3.6)	0.002
NT-proBNP (pg/mL)	1212.9 ± 1869.2 (8.4–8455)	1845.6 ± 2271.8 (4.1–9595)	0.089
LVEF (%)	28.93 ± 10.26 (16–43)	23.83 ± 7.04 (14–36)	0.012
SPAP (mmHg)	42.33 ± 19.03 (15–63)	45.25 ± 13.34 (22–70)	0.344

The results showed a mild significant correlation between the AST/ALT ratio and the following variables: APRI, FIB-4 score, NYHA-class, and LVEF (r = 0.2, 0.25, 0.26, and − 0.24;P < 0.05) respectively.

Additionally, LVEF had significant correlations (− 0.26, − 0.46, − 0.29, − 0.25; P < 0.05) with NYHA-class, left ventricular end systolic pressure, SPAP, and NT-proBNP, respectively.

**Fig. 1:**
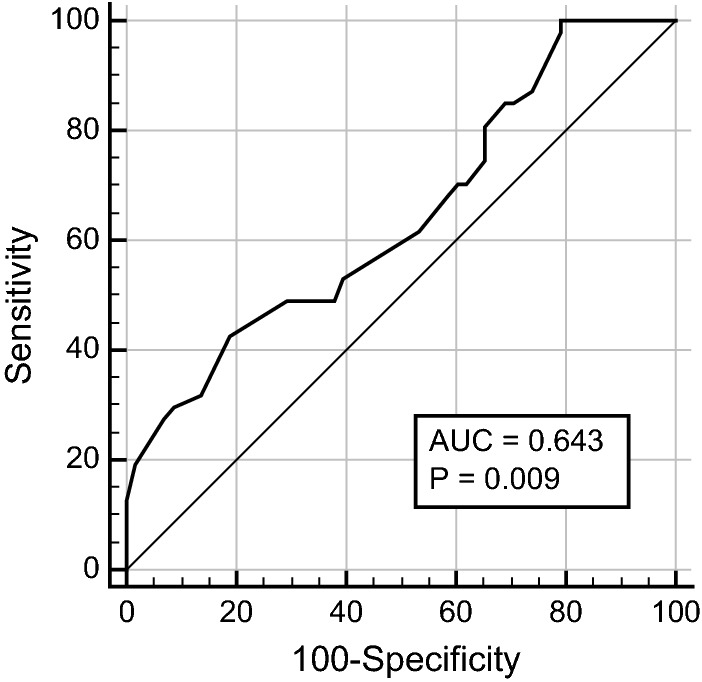
AROC-curve showing ALT/AST ratio prediction of reduced LVEF

The receiver operating characteristic (ROC) curve showed that the ASL/ALT ratio could predict the functional severity of LV systolic failure measured by LVEF. We used the American Society of Echocardiography cut-off value (LVEF < 30%) for diagnosing patients with severely impaired LVEF [19]. The area under the curve (AUC) was 0.64 (P < 0.05) with a 95% confidence interval of 0.54–0.73 with a best cut-off value of 0.9, sensitivity of 43.6%, and specificity of 81.4% (Fig. 1).

In our multivariate linear regression model that included age, body mass index (BMI), diabetes mellitus, hypertension, and NT-proBNP, the AST/ALT ratio was a significant independent predictor of LVEF% (95% confidence interval, 0.85–1.01; P < 0.05, Table 2).

**Table 2:** Multivariate linear regression model for prediction of LVEF(%)

Parameters	Standardized coefficient	95% CI	P-value
Age	− 0.174	47.25–52.94	0.102
BMI	0.061	27.80–30.99	0.696
Diabetes mellitus	0.433	1.56–1.75	0.879
Hypertension	1.903	1.46–1.66	0.502
AST/ALT ratio	− 6.796	0.85–1.01	0.045
NT-proBNP	− 0.001	932.91–2007.76	0.195

## Discussion

Our main finding in this study is that an increased AST/ALT ratio could predict functional status decline in HFrEF patients, as shown by the less favorable cardiac profile in Group-2.

Moreover, the predictive value of the AST/ALT ratio is independent after adjustment for age, BMI, hypertension, diabetes mellitus, and NT-proBNP.

Our explanation for this main finding is multi-factorial; it is known that AST is released from many tissues, including the myocardium and the liver, while ALT is only released from the liver. Therefore, more severe myocardial pathology would lead to an anticipated increase in the AST/ALT ratio [21]. Additionally, higher the AST/ALT ratio, greater the probability of hepatic fibrosis, which is associated with cardiovascular disease (CVD) pathogenesis through different mechanisms, including increased plasma inflammatory mediators, insulin resistance, oxidative stress, and metabolic syndrome [22].

Based on their medical records, our patients did not have any primary hepatic disorders to justify the increased AST/ALT ratio. It is known that an increased AST/ALT ratio is also caused by alcoholic liver disease [23]. However, our records did not include those with alcohol drinking habits.

Hypoxic hepatitis is another cause of increased AST/ALT ratio in its early phase; however, our data showed that ALT and AST values were far less than those required for diagnosing classic hypoxic hepatitis [24].

Previous research has focused on the value of hepatic transaminases as CVD predictors. Lazo et al. [25] showed that elevated levels of liver transaminases were significantly correlated with the cardiac biomarkers; troponin T and NT-proBNP. Consequently, they concluded that liver transaminases could be used as predictors in patients at risk of CVD. However, they did not enroll heart failure patients in their study, as they were only concerned with predicting subclinical myocardial injury.

Yokoyama et al. [26] recently concluded that an increased AST/ALT ratio positively correlates with NT-proBNP levels, yet their study was not specific for HFrEF patients.

Zoppini et al. [27] studied patients with type 2 diabetes mellitus and found that the AST/ALT ratio positively correlated with CVD mortality; however, as in the above studies, they did not include HFrEF patients.

On the other hand, there are some studies that did not show significant correlation between ALT and CVD, as seen by both Ruhl et al. [28] and Fraser et al. [29] However, they did not include AST or the AST/ALT ratio in their correlations with CVD, which could be an explanation for the non-significant result.

In our study, we found that 0.9 was the best predictive cut-off value of the AST/ALT ratio when considering functional severity in HFrEF patients. Our finding was in agreement with the finding of Long et al. [30] that 1 was the best cut-off value of the AST/ALT ratio that predicted cardio-metabolic risk in their study.

To our knowledge, our study is the first in Saudi Arabia and other Arabian countries to correlate the AST/ALT ratio with functional severity in HFrEF patients. Consequently, we could not find comparable AST/ALT cut-off values in the Arab population.

Our cut-off value could be very helpful in monitoring the functional status of HFrEF patients in primary healthcare settings and accordingly adjusting their follow-up investigations/management plans.

## Conclusion

Our study highlighted the value of the AST/ALT ratio as a simple independent predictor of LV functional status in patients with HFrEF.

## Recommendation

Based on our findings, we recommend future prospective studies to establish the potential practical value of AST/ALT ratio in monitoring the functional status of patients with HFrEF.

## Limitations

Our study had some limitations, including its retrospective nature; accordingly, we could not follow-up with the patients for detailed morbidity and mortality records in relation to AST/ALT ratio measurements. In addition, our study was a single-center study and prospective multicenter studies could improve our understanding regarding the correlation of AST/ALT ratio with HFrEF functional status.

## Data Availability

The datasets used and/or analyzed during the current study are available from the corresponding author on reasonable request.

## Abbreviations

ALT: Alanine aminotransferase
APRI index: AST to platelet ratio index
AST: Aspartate aminotransferase
AUC: Area under the curve
CVD: Cardiovascular disease
FIB-4: Fibrosis-4
HFrEF: Heart failure with reduced ejection fraction
LV: Left ventricular
LVEF: Left ventricular ejection fraction
LVEF (%): Left ventricular ejection fraction percentage
NYHA: New York Heart Association
NT-proBNP: N-terminal pro-Brain natriuretic peptide
ROC: Receiver operating characteristic
SPAP: Systolic pulmonary arterial pressure
